# Rare Co-Occurrence of Visual Snow in a Female Carrier With RPGR^ORF15^-Associated Retinal Disorder

**DOI:** 10.3389/fgene.2021.728085

**Published:** 2021-10-01

**Authors:** Aekkachai Tuekprakhon, Aulia Rahmi Pawestri, Ragkit Suvannaboon, Ketwarin Thongyou, Adisak Trinavarat, La-Ongsri Atchaneeyasakul

**Affiliations:** ^1^ Department of Ophthalmology, Faculty of Medicine Siriraj Hospital, Mahidol University, Bangkok, Thailand; ^2^ Nuffield Department of Medicine, Wellcome Centre for Human Genetics, University of Oxford, Oxford, United Kingdom; ^3^ Faculty of Medicine, Universitas Brawijaya, Malang, Indonesia; ^4^ Research Division, Faculty of Medicine Siriraj Hospital, Mahidol University, Bangkok, Thailand

**Keywords:** x-linked retinitis pigmentosa, visual snow, retinitis pigmentosa GTPase regulator gene, random X-chromosome inactivation, case report, inherited retinal disease

## Abstract

X-linked retinitis pigmentosa (XLRP), a rare form of retinitis pigmentosa (RP), is predominantly caused by mutations in the retinitis pigmentosa GTPase regulator (*RPGR*) gene. Affected males often present with severe phenotypes and early disease onset. In contrast, female carriers are usually asymptomatic or show stationary phenotypes. Herein, we reported an 8-year-old female carrier, a daughter of a confirmed RP father with *RPGR* mutation, with an early onset of progressive cone-rod pattern retinal dystrophy. Additionally, the carrier experienced visual snow-like symptom as long as she recalled. Ophthalmological examination showed the reduction of visual acuity and attenuation of photoreceptor functions since the age of 5 years. Further analysis revealed a heterozygous pathogenic variant of the RPGR gene and a random X-inactivation pattern. Although she harboured an identical RPGR variant as the father, there were phenotypic intrafamilial variations. The information on the variety of genotypic and phenotypic presentations in XLRP carriers is essential for further diagnosis, management, and monitoring of these cases, including the design of future gene therapy trials.

## Introduction

Retinitis pigmentosa (RP) (OMIM 268000) is a term describing a group of heterogeneous progressive inherited retinal diseases with diverse genetic inheritance patterns, including autosomal dominant (ADRP), autosomal recessive (ARRP), and X-linked (XLRP) (Ferrari et al., 2011). Although XLRP only constitutes for 6–17% of RP (Sharon et al., 2000), it usually manifests as the most severe phenotypes (Wu et al., 2010) with relatively early disease onset (Beltran et al., 2014). The symptoms often started in early childhood to adolescence, comprising rod-cone or cone-rod pattern retinal dystrophy (Zahid et al., 2013), including reduced night vision, constricted peripheral visual field, reduced visual acuity, or abnormal colour vision. Severe and rapid progression of retinal degeneration is often found in male patients (Genead et al., 2010; Wu et al., 2010).

XLRP is predominantly caused by mutations in the RP2 and RP3 loci, the latter of which is also known as the retinitis pigmentosa GTPase regulator (*RPGR*) gene. The expressed proteins, RP2 and RPGR, are localized in the connecting cilia between the inner and outer segments of the photoreceptors (Beltran et al., 2014; Megaw et al., 2015). RP2 protein plays role in transporting proteins from the Golgi complex to the primary cilium (Evans et al., 2010), while RPGR is involved in protein trafficking from the inner photoreceptor segment to the outer (Gakovic et al., 2011). The RPGR protein exists in several isoforms: RPGR^ex1-19^, which takes part in the development of photoreceptors, and RPGR^ORF15^, the retina-abundant form, which functions in maintaining mature photoreceptors (Sharon et al., 2000; Beltran et al., 2014; Megaw et al., 2015). Mutations in the *RPGR* gene contribute for 70–90% of XLRP (Megaw et al., 2015). To date, 350 mutations have been identified, with ORF15 being the hotspot accounting for two-third of all pathogenic mutations (Megaw et al., 2015). ORF15 is a highly mutable region due to its unusual purine-rich repetitive nucleotide composition, which results in atypical DNA conformations and reduced fidelity during sequence replication (Vervoort et al., 2000).

Due to the X-linked inheritance pattern, female carriers of XLRP with the *RPGR* mutations are mostly asymptomatic or show relatively stationary clinical courses due to the random X-chromosome inactivation in females, resulting in less severe clinical manifestations than affected males (Wu et al., 2010; Migeon, 2020). However, slow progressing mild-to-moderate RP phenotypes occasionally occur. Several studies reported clinical presentations in female XLRP carriers, including reduced visual acuity, ERG abnormalities in at least one eye (Comander et al., 2015), tapetal-like reflex, and pigmentary alterations (Genead et al., 2010). Nevertheless, to date, there is limited information of co-occurrence of other neuro-ophthalmic findings, such as visual snow, in XLRP patients and carriers.

Visual snow is a condition in which affected individuals constantly perceive tiny snow-like dots in the entire visual field. The presence of at least two of the following conditions, including palinopsia, entoptic phenomena, photophobia, or nyctalopia, in addition to visual snow could be classified as visual snow syndrome (Wood, 2020). The exact prevalence of visual snow in the general population is not well described, but a cohort study in the United Kingdom found the prevalence of visual snow and visual snow syndrome to be 3.7 and 2.2% out of 1,015 examined participants, respectively (Kondziella et al., 2020). This condition commonly appears in adulthood (29–50.6 years) with no specific gender preference (Kondziella et al., 2020; Puledda et al., 2020). Migraine and tinnitus are often considered as the comorbidity of visual snow/visual snow syndrome (Schankin et al., 2014). Phosphene, light sensations without an actual light source, is a similar condition to visual snow (Császár et al., 2016). However, unlike visual snow that occurs persistently, phosphene is transient and usually co-occur with other ophthalmological conditions, including increased eye pressure, posterior vitreous detachment, diabetic retinopathy, or ocular migraine (Yildiz et al., 2019). In RP, phosphene was reported in 66% of the patients and appeared to be related with increased stress and severity of RP (Bittner et al., 2011; Bittner et al., 2012). The information of the variety of clinical spectrum in XLRP could enrich the disease.

Although currently there is no effective treatment for patients with XLRP, gene therapy could be a promising platform to decelerate or even halt the disease progression. With a relatively small gene size of 3.5 kb, gene therapy for this RPGR^ORF15^-associated disorder is promising yet challenging due to its repetitive and purine rich regions, since they are difficult for the plasmid manipulation processes (Martinez-Fernandez De La Camara et al., 2018). Several phase I/II clinic trials involving adeno-associated viral vectors to deliver the healthy *RPGR* gene are underway, including NCT03116113, NCT03252847, NCT03316560, and the initial result of the first-in-man clinical trial has met the safety criteria (Cehajic-Kapetanovic et al., 2020). Currently, most gene therapy trials focused on male XLRP patients. However, as some female carriers present prominent phenotypes, affected carrier should also be included in XLRP gene therapy trials (Nanda et al., 2018; Talib et al., 2018). One of the most advanced phase III trial (NCT04671433) is now recruiting participants with confirmed XLRP mutation regardless of gender. Data on genotype-phenotype correlation of XLRP could benefit the gene therapy clinical development in future clinical trials.

This study presented the unique co-occurrence of progressive cone-rod pattern retinal dystrophy and visual snow-like presentation in a young female XLRP carrier. The information could enrich the variety of XLRP forms and assist practitioners in proper detection, management, follow up, and counselling of these patients. Reports of disease phenotypes in carriers also aids in suggesting the inheritance pattern of this disease.

### Case Description

**FIGURE 1:**
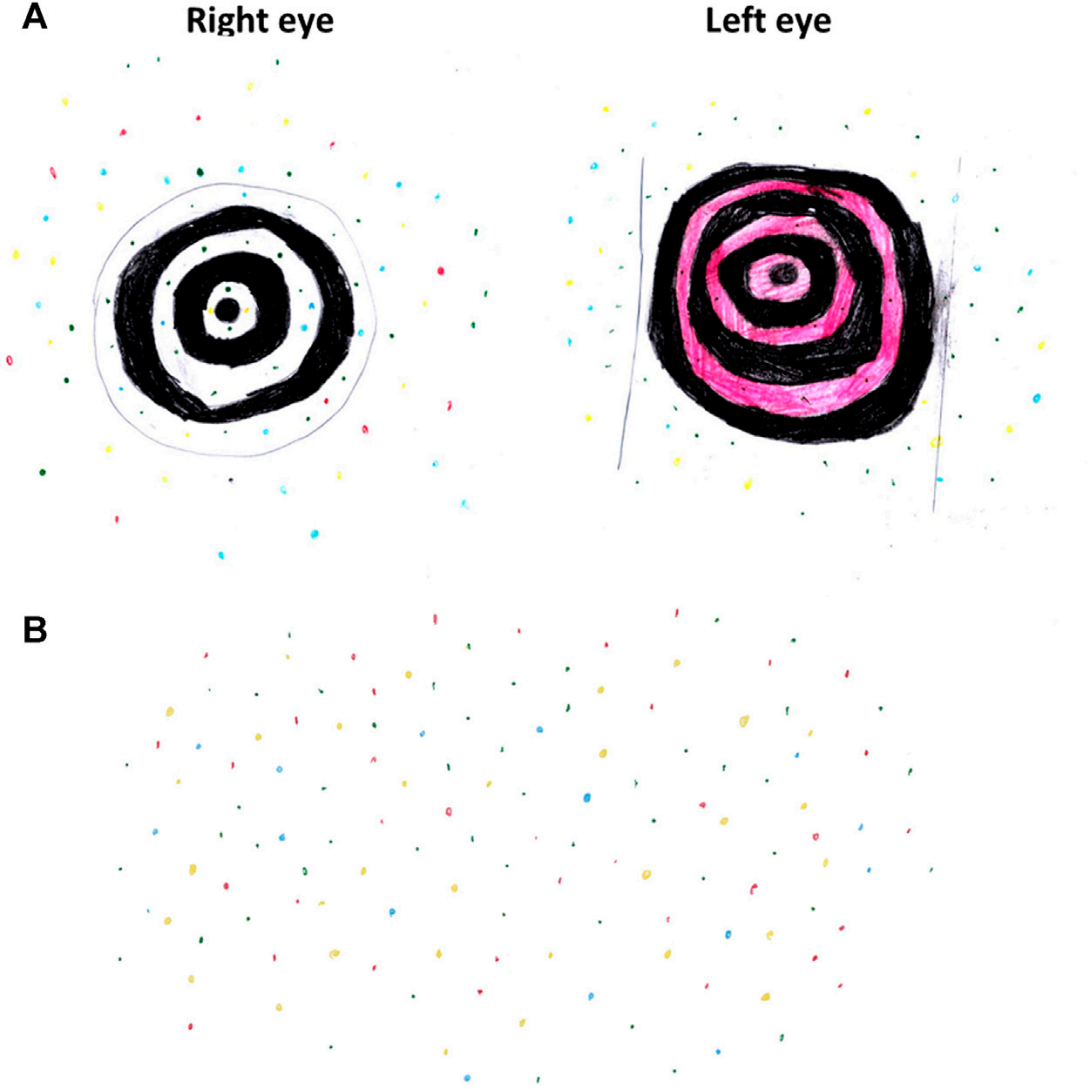
The patient’s drawing at 8 years old describing her visual experience. **(A)** Black-and-white or coloured swirls with colourful dots. **(B)** Colourful dots involving the entire visual field.

The patient was an 8-year-old female. The parents and patient had provided their written consent prior to enrolment in this study. The patient first came for ophthalmological evaluation at the age of 5 years due to the complaint of blurred vision. Her parents found out that she had been seeing “pretty moving colourful dots” and “swirling black-and-white or coloured circles” since she was 2 years old (Figure 1).

**FIGURE 2:**
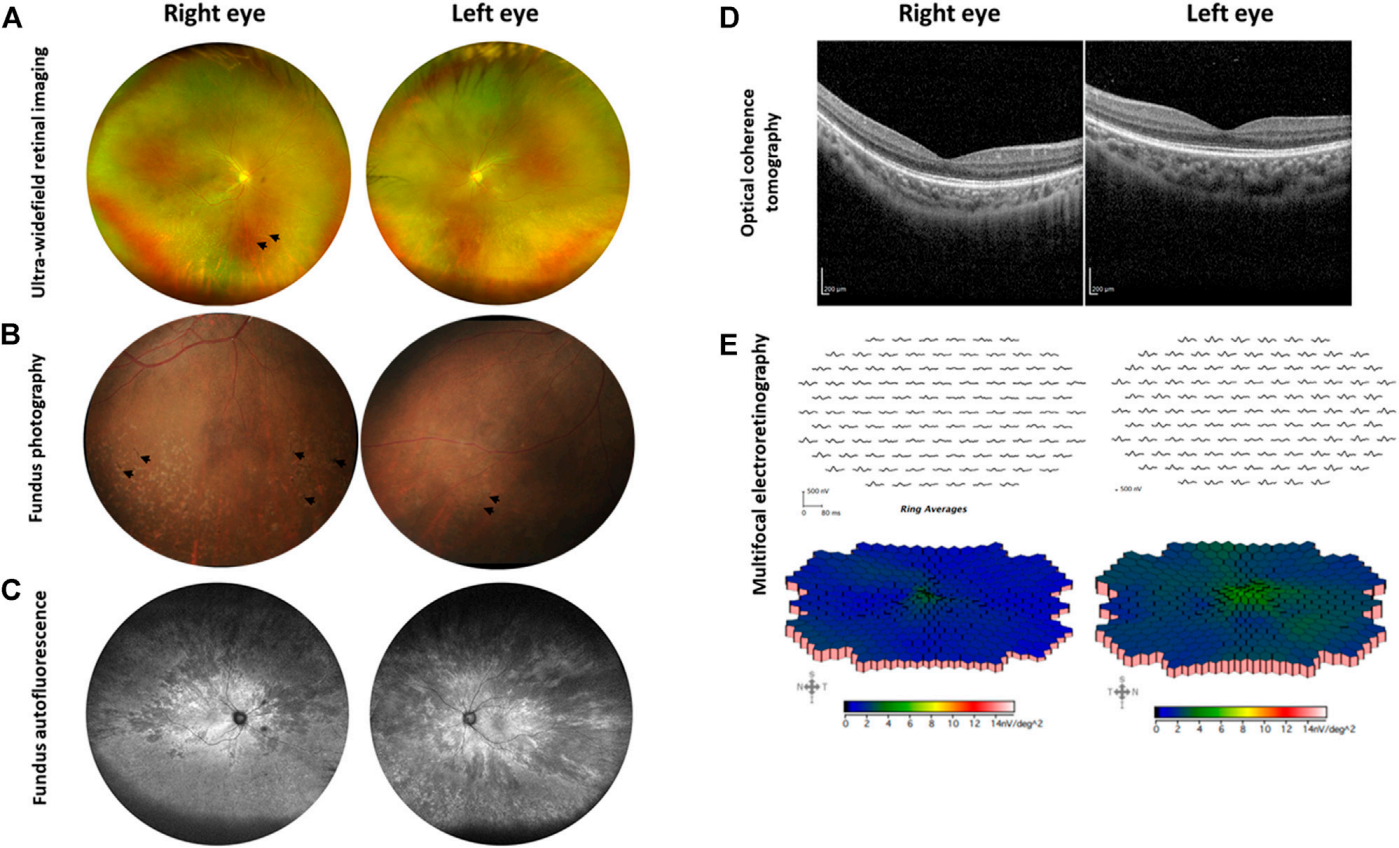
Ophthalmological examination of the XLRP carrier. **(A)** Ultra-widefield retinal imaging reveals pigmentary changes in the right eye as indicated by arrows, while the left eye shows milder changes. **(B)** Fundus photography reveals bone spicules in the inferior retina of both eyes, as marked by arrows. **(C)** Ultra-widefield fundus autofluorescence shows multiple patchy hypofluorescent areas distributing from the macula to the peripheral retina in both eyes. **(D)** Optical coherence tomography reveals preserved central foveal ellipsoid zones, with attenuation in the perifoveal area. **(E)** Multifocal electroretinography shows marked attenuation of the entire macular area of the retina (right eye) and diagonally patchy appearances of low amplitude responses (left eye). Data were retrieved from the ophthalmological examination at the age of 8 years.

At the age of 5 years, the patient had slight reduction in the best corrected visual acuity (BCVA) with Snellen acuity of 6/12 and 6/24 in the right and left eye, respectively, and mild reduction in the superior and inferior visual fields, while the colour vision was normal. The refraction showed mild myopia with moderate to high astigmatism in both eyes (right eye: −2.00−2.75@180, left eye: −1.25−6.25@170). She did not experience nyctalopia. Regarding the cone function, the electroretinogram (ERG) showed 38 and 7% reduction of flicker amplitude in the right and left eye, respectively. For the rod function, scotopic ERG showed 12% reduction of the amplitude in the right eye and normal amplitude in the left eye. The fundus photographs displayed generalized retinal pigment epithelium (RPE) changes in both eyes. Over the course of 3 years, we noticed the improvement of BCVA to 6/9 and 6/7.5 in the right and left eye, respectively (probably due to better cooperation of the patient) and stable visual field. However, she developed bone spicule pigmentation and white flecks at the inferior retina in both eyes, which were more prominent in the right eye (Figures 2A,B). Widefield fundus autofluorescence images revealed multiple patchy hypofluorescent areas distributing from the posterior pole to the peripheral retina in both eyes indicating loss of RPE cells (Figure 2C). Optical coherence tomography (OCT) showed attenuation of the ellipsoid zones in the perifoveal area, with well-preserved central fovea (Figure 2D). The ERG at the age of 8 years showed 57 and 42% reduction of cone function in the right and left eye, respectively. The rod function was minimally reduced in the right eye and remained normal in the left eye. The multifocal electroretinogram (mfERG) demonstrated marked attenuation of the entire macular area of the retina in the right eye and patchy low amplitude responses in the left eye (Figure 2E).

At the age of 8 years, her blood sample was subjected to mutation analysis (Molecular Vision Laboratory, MVL, OR, United States) using the MVL Vision Panel v7 to screen for identified pathogenic variants and variants of uncertain significance (VUS). The reading covered all the coding regions and immediately flanking intron sequences of 936 vision-related genes and the mitochondria genome. The mutation analysis revealed that the patient carried a heterozygous pathogenic variant with a frameshift mutation of amino acid 809 in the *RPGR* gene, NM_001034853.2: c.2426_2427delAG (p.Glu809GlyfsTer25) (ClinVar: pathogenic; two stars), resulting in a truncated RPGR^ORF15^ protein. The mutation caused a complete deletion of the basic domain, and most of the glutamic-acid/glycine-rich domain were replaced by 24 amino acids residues, resulting in changes in the protein’s isoelectric point (PI) from 4.23 to 4.68 (determined with the online Expasy compute pI/Mw tool, available at https://web.expasy.org/compute_pi/). The X-chromosome inactivation study (Greenwood Genetic Center, SC, United States) was conceived using the HUMARA assay based on the androgen receptor (AR) locus. The results revealed a ratio of 68:32, indicating a random X-inactivation pattern.

**FIGURE 3:**
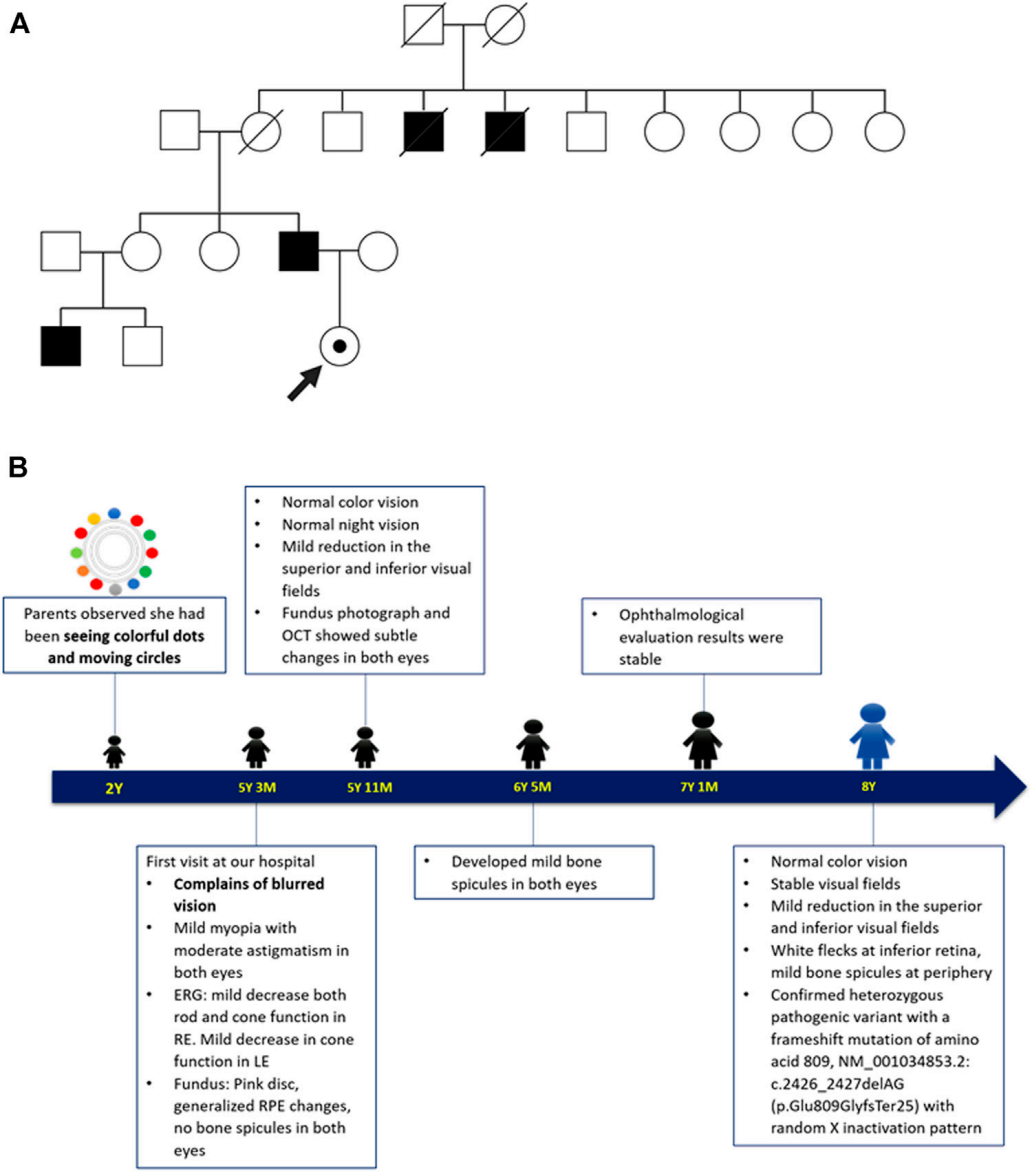
**(A)** The patient’s family pedigree. The pedigree was drawn based on the father’s description on their family members presenting symptoms suggestive of XLRP. Circles indicate female and squares indicate male. Close symbols indicate family members with symptoms and open symbols indicate unaffected individuals. The dotted circle indicates the patient as a female carrier. The strike-through symbols indicate deceased family members. **(B)** Timeline of patient care. The patient’s parents first noticed her seeing colourful moving dots, possibly representing visual snow, at the age of 2 years. She first came for ophthalmological evaluation at the age of 5 years due to blurred vision. The follow up was performed five times over the course of 3.5 years, where the patient displayed significant reduction of the cone function in both eyes. The mutation analysis and the X-chromosome inactivation study were performed at the age of 8 years.

The patient was the only child of an affected male with XLRP. Although they shared the same mutation, their phenotypes demonstrated an intrafamilial variability. The father showed the onset of rod-cone pattern RP (rod-dominated disruption) at his teenage years, while the patient presented with cone-rod pattern retinal dystrophy (cone-dominated disruption) since her childhood. At the age of 35 years, the father was unable to read and ophthalmological examination at the age of 41 years revealed visual acuity of hand motion and generalized RPE changes with moderate bone spicules in both eyes. The father also reported nyctalopia in two maternal uncles and the son of his elder sister. The detail of the pedigree and timeline of the patient’s follow up is as shown in Figures 3A,B.

The patient and family were hoping for a definitive treatment. Unfortunately, only supportive treatment is available to date. We prescribed suitable glasses throughout her visit. Furthermore, genetic counselling was provided for the patient and family. We have also informed them of the ongoing clinical trials for their condition. Although the patient developed abnormal cone function since childhood, long term follow up is required to determine visual deterioration since a significant number of RP patients demonstrated stable vision for several decades.

## Discussion

Herein we reported a young female XLRP carrier of a pathogenic variant in the *RPGR* gene with an early onset of abnormal cone function, bone spicules, and visual snow-like symptom. At 5-year-old, she started showing signs of cone dysfunctions and slight abnormalities in the retina. 3 years later, there were progressive deterioration in the cone functions and more prominent changes in the retina. Fortunately, she was still able to perform her daily activities without any significant disturbances.

Our patient reported continuously seeing colourful dots and spirals in her entire visual field for as long as she could remember. This symptom closely resembled the pulse type visual snow, which is described as flickering dots scattered across the visual field (Yoo et al., 2020). Visual snow is linked to a hyperexcitability state in the brain cortex and thalamus (Metzler and Robertson, 2018). Although we still could not elucidate whether the visual snow-like symptom is correlated or just co-occurring with the XLRP, we affirmed that our patient did not have history of migraine, head trauma, or seizure. It is noteworthy that closely resembling conditions, including phosphene or flashing lights, are possible differential diagnoses (Yildiz et al., 2019). Nonetheless, in our case, the patient complained of persistent dot-like appearances in her entire visual field with a very early onset, whereas phosphene is usually transient and worsen with the deterioration of RP (Bittner et al., 2011; Bittner et al., 2012). Thus, visual snow was a suitable diagnosis in this patient although further investigations, including electroencephalogram and brain CT, may elucidate the aetiology of the visual snow-like symptom.

**FIGURE 4:**
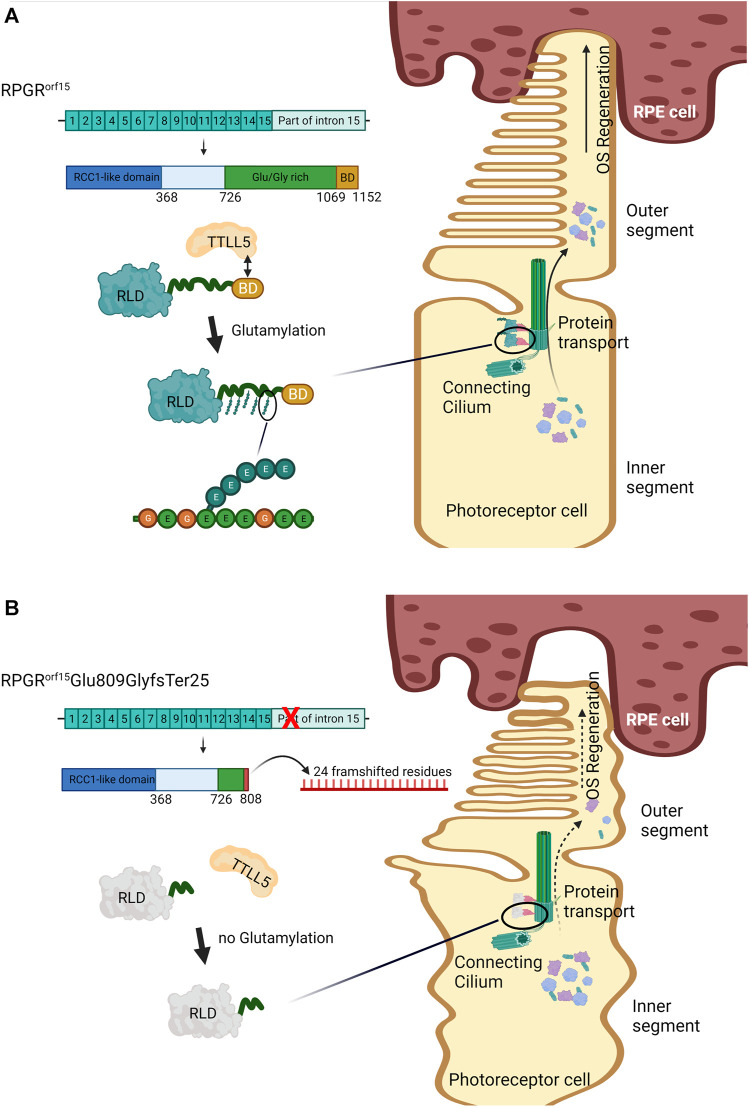
Effect of the glutamylation on the RPGR^ORF15^ wildtype and RPGR^ORF15^Glu809GlyfsTer25. **(A)** The wildtype RPGR^ORF15^ protein uses the basic domain (BD) to interact with Tubulin Tyrosine Ligase-Like 5 (TTLL5). The TTLL5 induces glutamylation to the repetitive amino acid sequence on the Glu/Gly-rich region at the C-terminus of RPGR^ORF15^. The glutamylated RPGR^ORF15^ at the connecting cilium performs its role in protein trafficking, allowing effective transportation of opsins and other proteins from the inner (IS) to the outer segment (OS), which is critical for the regeneration of the OS and photoreceptors survival. **(B)** The mutant RPGR^ORF15^Glu809GlyfsTer25, lacking the BD and most of Glu/Gly-rich region, could not interact with TTLL5 and undergo glutamylation. The mutant performs poorly on protein transportation, impairing the OS regeneration and photoreceptor vitality. The image was created with BioRender.com. BD: basic domain, OS: outer segment, RCC-1: regulator of chromosome condensation 1, RLD: RCC1-like domain, RPE: retinal pigment epithelium, RPGR: retinitis pigmentosa GTPase regulator, TTLL5: Tubulin Tyrosine Ligase-Like 5

Under normal circumstances, *RPGR^ORF15^* encodes a protein with a unique glutamic-acid/glycine-rich domain and a basic domain (BD) in the C-terminus. This protein was primarily found in the connecting cilium, a gateway for protein transport of the photoreceptors (Figure 4A). Although the function of the Glu/Gly-rich domain is still unclear (Beltran et al., 2014; Megaw et al., 2015), most pathogenic mutations of XLRP is found in this sequence, mainly as frameshift mutations producing truncated RPGR proteins with loss of the BD (Figure 4B) (Megaw et al., 2015), as occurring in our case. The BD was reported to interact with Whirlin (WHNR), an Usher syndrome-related protein that plays role in regulating protein transport (Wright et al., 2012). As shown in Figure 4A, the BD plays an important role in the glutamylation, a posttranslational modification, of the RPGR^ORF15^ Glu/Gly-rich domain by interacting with the Tubulin Tyrosine Ligase-Like 5 (TTLL5) (Rao et al., 2016; Sun et al., 2016). It was reported that glutamylation in other proteins, such as tubulin, regulates the activity of molecular motors (Janke et al., 2008). Thus, presumably, the glutamylation of the RPGR^ORF15^ is also associated with the transportation mechanism in the connecting cilium (Figure 4A). The absence of this posttranslational modification in the RPGR protein diminish the protein function and is associated with retinal diseases (Megaw et al., 2015). Animal studies found that the *Ttll5* mutant mouse presented the same disease phenotype as the *Rpgr-null* mice (Sun et al., 2016), with an absence of glutamylation in the RPGR^ORF15^ mutant (Rao et al., 2016), indicating the critical necessity of the glutamylation on the RPGR^ORF15^.

The RPGR variant found in our patient had an increased isoelectric point (pI) and expressed a noticeable phenotype. This phenomenon is similar to the animal model where the frameshift mutation in ORF15 with an increased pI (from 4.01 to 4.30) showed evidence of protein aggregation and more severe phenotypes than immediate stop codon mutations with minimal pI change (Beltran et al., 2014). We hypothesize that frameshift mutations with increased pI might alter an essential biochemical property of the ORF15 by deranging its negative charge, which in turn results in a more severe phenotype.

Phenotypic differences in the same *RPGR* mutation have been reported. Cone- and rod-dominated phenotypes were found to shift between two mice strains with the same *Rpgr* mutation (Brunner et al., 2010). In human, rare occurrences of intrafamilial phenotypic variation were documented. Although sharing an identical mutation, one family member showed cone-rod dystrophy (OMIM 304020), while the other expressed a phenotype of RP (Walia et al., 2008; Talib et al., 2018). Our female carrier presented different phenotypes from her father despite harbouring an identical pathogenic frameshift mutation in the ORF15. She displayed decreased cone functions at an earlier age and slight attenuation in the perifoveal ellipsoid zones, which are mostly populated by cone cells, with minimal reduction of rod function. On the other hand, the father showed disease onset in his teens, starting with rod dysfunction, and further progressing to cone deterioration. Similarly, a study in Japan reported an intrafamilial variability in the same *RPGR^ORF15^* mutation as our patient. The proband (father) showed early onset of typical XLRP with rod-cone dystrophy pattern at the age of 10 years, while his daughter, an XLRP carrier, presented with myopia and non-responsive ERG since the age of 3 years (Jin et al., 2006). This varying phenotype might be influenced by genetic modifiers, where cone-specific modifiers play role in the cone-rod pattern, and vice versa. A study attempting to identify possible RPGR-associated genetic modifiers found that single nucleotide polymorphisms (SNPs) in RPGR-interacting proteins, such as RPGRIP1-like (RPGRIP1L) and IQ motif containing B1 (IQCB1), were statistically associated with the disease severity (Fahim et al., 2011). Further research is required to reveal the molecular mechanism and effects influenced by genetic modifiers. Another potential explanation for phenotypic differences includes the X-chromosome inactivation in female carriers, which was said to play crucial role in varying phenotypes compared to the male patients (Migeon, 2020).

The unique finding in our carrier patient is the early formation of bone spicules. To date, there are no reports on bone spicule development in female XLRP carriers of this mutation. However, to confirm our unique finding and the possible genotypic-phenotypic correlation, more reports on the carriers and probands of this mutation are required.

The random X-inactivation pattern was confirmed in our patient. This result was in accordance with the patchy pattern of retinal degeneration and asymmetric pathology in her left and right eye, which was possibly due to cell mosaicism. Similar observation was reported in heterozygous animal models with frameshift mutation in the ORF15 (Beltran et al., 2014; Wood, 2020). Female carriers are usually less affected in X-linked diseases (Migeon, 2020), provided that the normal cells can compensate for the defective cells and express the normal phenotype. However, some mutations in the X-linked gene show the dominant form by expressing the disease phenotype in heterozygous females. A study in 141 families with RP in Switzerland showed a variety of phenotypes in female carriers of *RPGR^ORF15^* mutations ranging from asymptomatic to severe clinical presentations. One of the mutations, c.2405_2406delAG, was assumed to have dominant inheritance and the two affected female carriers in the family showed mild and severe clinical outcomes, respectively (Neidhardt et al., 2008). The skewness of X-inactivation can influence the severity of phenotypes in female carriers with X-linked diseases (Franco and Ballabio, 2006). The early and progressive presentation of our patient in combination with the random X-inactivation pattern suggested the possible dominant inheritance of the c.2426_2427delAG mutation. Similar phenotypes had been reported in a study in Japan (Jin et al., 2006). Nevertheless, the assumption of a dominant mode of inheritance requires more investigations of the phenotypes in other carriers for this mutation to achieve a more conclusive verdict.

Through extensive gene screening, we have excluded other coexisting genetic causes. Moreover, the patient was routinely monitored to observe the disease progression. Unfortunately, since the parents did not immediately seek medical testing by the time the patient first exhibited symptoms, there might be recall bias regarding the exact age of onset. Since comprehensive neurological testing was not performed on this patient, other differential diagnoses of the visual snow-like symptom could not be completely excluded. It is also important to note that peripheral blood sample might not be the best representation for X-chromosome inactivation studies of the retinal cells due to the random process of X-inactivation in individual cell types (Banin et al., 2007; Nanda et al., 2018).

In summary, we reported the unique co-occurrence of an early onset, progressive cone deterioration with bone spicule formation and visual snow-like symptom in a female XLRP carrier harbouring *RPGR* mutation (p.Glu809GlyfsTer25). Nevertheless, the information on the wide spectrum of genotypic and phenotypic presentation in XLRP carriers is essential for further diagnosis, management, and counselling of these cases. Moreover, additional reports on the disease progression and clinical findings in carriers of this *RPGR* mutation could aid in the considerations for the design of gene therapy clinical trials in the future. Long-term follow up of the carriers can also help in suggesting the inheritance pattern of the disease.

## Data Availability

The original contributions presented in the study are included in the article/Supplementary Material, further inquiries can be directed to the corresponding author
